# Credibility: A metric for graduate medical education evaluation

**DOI:** 10.1016/j.acpath.2026.100303

**Published:** 2026-10-15

**Authors:** Thomas J. Cummings

**Affiliations:** Duke University Medical Center, Department of Pathology, Durham, NC, USA

**Keywords:** Credibility, Graduate medical education, Mentorship, Trust

## Abstract

Credibility is the quality or power of inspiring belief. Credibility has an important role in science and medicine, including relations with the press; physician-industry relations; and, public trust, mistrust, and perception. Residency and fellowship program directors seek to best prepare trainees for postgraduate medical education careers, and nurturing credibility is one goal to strive for. Credibility is different from trust. Credibility is a subjective evaluation, in contrast to objective criteria evaluated in entrustable professional activities. The goal of this manuscript is to introduce consideration of credibility as a metric in Graduate Medical Education evaluations, and as a discussion topic during mentorship of trainees.

## Introduction to credibility and graduate medical education

The Accreditation Council for Graduate Medical Education (ACGME) uses Milestones to assess and define competency-based medical education. Milestones integrate with the six core competencies: Professionalism; Patient Care and Procedural Skills; Medical Knowledge; Practice-Based Learning and Improvement; Interpersonal and Communication Skills; and Systems-Based Practice.^1^^,^^2^ Entrustable professional activities (EPAs) are another metric to evaluate trainees (residents and fellows).^3^^,^^4^ Entrustable professional activities evaluate the trustworthiness of the trainee to complete tasks. Combined data from Milestones and EPAs provide Clinical Competency Committees (CCCs) with information to evaluate Graduate Medical Education (GME) performance. The ultimate outcome of GME is excellence in resident education and patient care.^5^ To help achieve this, ACGME Program Requirements for GME in Anatomic Pathology and Clinical Pathology state, “There must be one faculty member appointed as program director (PD) with authority and accountability for the overall program, including compliance with all applicable program requirements” (Section 2: Personnel; 2.1); and, the PD as the leader of the program, must serve as a role model to residents in addition to fulfilling the technical aspects of the role. As residents are expected to demonstrate compassion, integrity, and respect for others, they must be able to look to the PD as an exemplar (Section 2: Personnel; 2.6a).^5^ Program directors, therefore, must strive to attain a high level of credibility, and to promote these traits for GME trainees to model and aspire to.

Program directors, CCCs, faculty, and institutional GME leaders work together to educate, mentor, and sometimes remediate trainees. Mentorship, continued education, and remediation may also benefit physicians who have successfully completed GME training. Remediable examples may include medical knowledge and professionalism, addiction, medicolegal situations, criminal behavior, and others. These can result in diminished trustworthiness and/or credibility.

## Credibility and trust: Definitions and differences

The Merriam-Webster dictionary (merriam-webster.com) defines credibility as the quality or power of inspiring belief, and defines trustworthiness as worthy of confidence, dependable. Although the definition and interpretation of credibility and trustworthiness may overlap, credibility can be parsed from trustworthiness and therefore has potential to serve as a metric for evaluation and mentorship of GME trainees. Four relationships exist between credibility and trust: An individual can be credible and trustworthy; credible and not trustworthy; trustworthy and not credible; and, not trustworthy and not credible. Physicians strive to be credible and trustworthy. Graduate Medical Education leaders strive to assist trainees who localize to one of the other three categories, with the goal for the trainee to improve to the desired level of being considered both credible and trustworthy.

Trustworthiness is an assessment from an objective, informed perspective, whereby the PD and CCC evaluate objective data, including EPAs, to assess if the subject is reliably achieving ACGME Milestones and specialty competency. Credibility is a subjective, involved impression. It is perhaps formulated by professional colleagues or patients, i.e., opinions about whether an action or communication is reliable (consistent and dependable over time) and competent, but it is not formulated by the defined milestones and EPA standards. Thus, the distinction between trustworthiness and credibility is evident. If an individual is credible, we “believe” in them. Credibility is an attribute that others invest in you, and thus it is a slippery concept, in that feelings of credibility arise from your own perceptions of that external investment; essentially, we seek to maintain an attribute that is always given to us by other people. Patients select their physicians, in part, based upon their research and subsequent subjective opinion of a potential physician's credibility. Therefore, because credibility is subjective, can it be remediated, coached, and/or mentored? Perhaps there is a role to integrate a discussion of credibility into the evaluation, remediation, and mentorship processes to benefit trainees as we strive to assist them to develop the traits that may ultimately affect their own credibility.

## Credibility and trust: Cautionary tales

Credibility plays an important role in learning, teaching, and in clinical practice. Tension between expressing vulnerability and seeking credibility creates challenges for learning and teaching, particularly in health care, in which practitioners regarded as credible may make an error that leads to dire consequences and blame. The compelling act of candor in teaching and learning can be dashed abruptly when credibility is lost, and it is suggested that learners and teachers alike should embrace the ongoing struggle between expressing vulnerability and seeking credibility, both in learning and in practice.^6^ Caution, however, about trustworthiness may also be warranted. While the rhetorical conception that high levels of interpersonal trust contribute to improved communication practices, trustworthy communication may enable a receiving physician to take some things for granted, to use communicative shortcuts, and to require less confirmatory questioning, and constitute a threat to patient safety.^7^^,^^8^ These cautionary tales are reminiscent of gain, loss, and protection of one's reputation.

## Credibility: Benefits in medicine and education

Publications in *Science*,^9^ *Scientific Reports*,^10^ *New England Journal of Medicine*,^11^ and *Proceedings of the National Academy of Science USA*^12^ advocate for credibility as it relates to science and medicine, including relations with the press; physician-industry relations; and public trust, mistrust, and perception (e.g., retracted publications from alleged falsification of data compromise credibility). We want physicians to be competent, trustworthy, and credible. As we want those in other professions; e.g., to name a few, pilots, mechanics working on aircraft and vehicles, nurses, pharmacists, law enforcement, educators, and countless others. Reports of the benefits of credibility exist in business, nursing, medical education, and leadership. In the business sector, character (integrity), competence (skills and results), and caring (kindness and concern) are the components of credibility used in organizational and leadership communication, and build off Aristotle's introduction to credibility (ethos) in his work *Rhetoric*.^13^ Learners in the nursing profession in educational and leadership programs report that the enhanced sense of credibility increases their professional and personal confidence and resilience to overcome challenges to complete the education program,^14^^,^^15^ especially when educators are facilitators of trust.^16^

## Credibility: A proposal to evaluate and mentor GME trainees

A *New England Journal of Medicine* (NEJM) advertisement for the Editor-in-Chief (EIC) of *NEJM Clinician* seeks an EIC who brings national recognition, clinical credibility, and a trusted public voice to a new, fast-growing publication for clinicians, and the minimum qualification includes “demonstrated credibility with practicing clinicians through academic leadership, widely read publications, or national service.”^17^ A goal for PDs, educators, and mentors, therefore, is: Can we assist a GME trainee to attain the required level of credibility to earn a position such as this? Is the answer innate in residents and fellows? If not, can credibility be nurtured during GME training? Can it be evaluated? Can it be a metric incorporated into Milestones and/or EPAs? Or is credibility an obvious and nonremediable characteristic that a GME physician trainee either “has it and gets it” at a young stage in their career, or they “don't have it and they don't get it,” – “it” being the level of trust, integrity, and professionalism – or, character, competence, and caring - wrapped up in credibility.

One proposal is that a discussion about credibility can be introduced during one-on-one meetings between PDs and trainees, in CCC meetings; and in letters of recommendation and/or references for fellowships and post-GME practice. When meeting with trainees, perhaps credibility can be introduced as a quality that can be gained (over time), lost (sometimes abruptly), compromised or undermined. One example for discussion could be how GME leaders can build credibility and model a growth mindset by admitting mistakes and apologizing (if appropriate),^18^ in contrast to the opposite approach of never admitting a mistake and never apologizing. When our trainees leave GME, their prospective employers can benefit during the recruitment process if the PD serves as a reference and confirms the trainee displayed a high level of credibility during residency, a subjective metric to complement the objective criteria assessed by Milestones and EPAs. Program directors, educators, teachers, coaches, and mentors seek to best prepare our trainees for their post-GME careers. Entrustable professional activities address the graduated responsibilities of a categorical GME training program, which are the building blocks of the competency component of credibility.

Caution is warranted not to concatenate objective graduated competencies and responsibilities of becoming a competent physician in the categorical specialty with the absolute requirements for character, i.e., subjective credibility, which apply at all levels of training. Perhaps an introductory first step to the evaluation of credibility is to include the word credibility as a subjective assessment item in EPAs, and to allow for further discussion if expansion of the assessment of credibility as a metric for evaluation is possible.

## Credibility, ACGME Milestones, and EPAs

Does documentation of credibility have a role in the evaluation of trainees? Literature to support this topic is lacking. A search of the word “credibility” in the *Journal of Graduate Medical Education* search bar identifies more than one hundred references, although none of them align with the goal of this manuscript. A cohort of the references, however, does report the issue of credibility from the viewpoint of trainees; that is, trainees may perceive that their professional credibility could be at risk when they interact with faculty, i.e., if they ask for help or choose not to ask for help during a range of clinical situations^19^ and in the context of when feedback takes place.^20^ If residents are concerned about their own credibility, this perhaps supports the premise of this manuscript that we, their advisors, mentors, and PDs should also be concerned.

In the GME setting, trust and integrity (character) are components of professional credibility that must be operative at all times. Perhaps these standards are not subject to gradation, and one cannot be partially trustworthy, or partially committed to assigned responsibilities in patient care, as a graduated work-in-progress toward a higher level of trustworthiness and commitment. If there is a violation of these absolute standards, PDs and the institution will have to consider the remediation and corrective actions required to restore the trainee to good standing. To the extent that the trainee cannot successfully remediate, determination must be made for the trainee's eligibility to complete their program. State Medical Boards offer rehabilitation and re-entry programs for issues of trust and integrity in licensed practitioners; the same considerations can apply within a given GME training program. The “Correctional System” is named as such to emphasize that rehabilitation is the goal to help improve offenders. A most rewarding aspect of the PD role is the successful remediation of a trainee that results in personal growth, competency, and perhaps an increase in their credibility. If there is a pathway forward for remediation, perhaps credibility can then be considered for evaluation of improvement. Trainees enter residency at a level of credibility built during medical school and other experiences. Our goal is to mentor each trainee to a higher level of credibility by the time they graduate residency, and allow the foundation built during residency to continue to strengthen during fellowship and post-GME practice. Figure 1 illustrates how the foundational qualities of character (do what is right), competence (do what you do well), and commitment (care and concern about others) align with the ACGME Program Requirements and work together to build the foundation of trustworthiness (honesty), which then earns credibility (inspires confidence), and how the achievement of credibility results in a continuous cycle that reinforces trust.

**Fig. 1:**
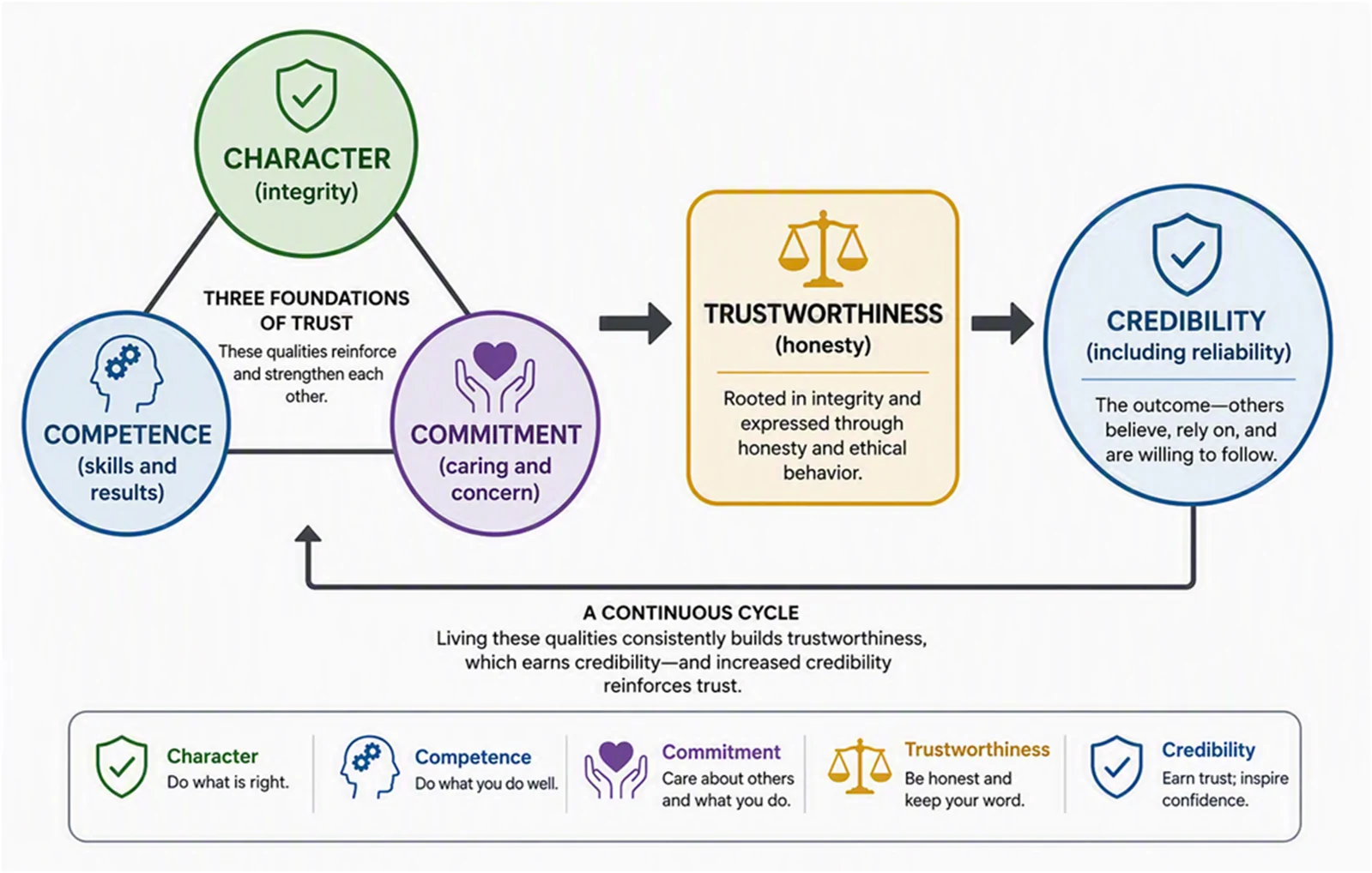
Schematic showing the conceptual relationships between character (integrity), competence (skills and results), and commitment (caring and concern); trustworthiness (honesty); and credibility (including reliability).

Despite its subjective nature, can the degree/level of judgment of credibility align/map to ACGME Milestones? A proposal is offered (Table 1) to align credibility with ACGME Milestones and EPAs for Anatomic and Clinical Pathology residency. Evaluation of credibility can then be introduced into summative evaluations (Table 2). It is reasonable that these proposals can be tailored and adapted for Milestones and EPAs in other residency specialties.

**Table 1:** Proposal to align credibility with ACGME Milestones and EPAs for Anatomic and Clinical Pathology residency.

Level 0: The resident enters graduate training at a foundational level of credibility, informed by experiences gained in undergraduate education, medical school, employment, community involvement, and other experiences. The inviolable personal characteristic of honesty and commitment to patient care must always be operative. The resident and the training program then strive to increase the level of credibility during residency in preparation for fellowship and beyond.
Level 1: The resident begins to acquire additional traits of credibility. The resident is teachable and enthusiastic to learn; is receptive to feedback; writes preliminary autopsy and surgical pathology reports and transfusion reactions on time; and adjusts efficiently to the residency internship year.
Level 2: The resident builds on Level 1, appropriate to the level of training, and demonstrates involvement in academic work, e.g., prepares an abstract for presentation at a local or national meeting; presents cases at tumor boards; seeks opportunities for graduated responsibility; and displays an appropriate level of confidence aligned with skill.
Level 3: The resident builds on Level 2, appropriate to the level of training, and continues to show increasing reliability and trustworthiness, and increased involvement in academic work, e.g., first-author publication and/or takes the lead in a research project; delivers a high-level Grand Rounds presentation.
Level 4: The resident builds on Level 3, appropriate to the level of training, and demonstrates continued improvement to achieve credibility, at or above the level of training. Upon completion of residency, the resident has achieved a level of credibility that is justifiable and worthy of inclusion in a letter of recommendation.

**Table 2:** Examples of evaluations for a level 4 resident: appropriate credibility for level of training (1); and, subpar credibility for level of training (2).

1. Appropriate credibility for level 4 resident: I worked with Dr. Resident for two weeks during their molecular integration rotation. We examined a high volume of challenging surgical pathology cases. Dr. Resident understood key points of the World Health Organization diagnostic histology and molecular correlation and classification; prepared excellent integrated final reports that required minimal editing; and, consistently displayed exemplary medical knowledge, professionalism, reliability, and trustworthiness. This fourth-year resident exhibits credibility expected for their level of training.
2. Subpar credibility for level 4 resident: I worked with Dr. Resident for two weeks during their molecular integration rotation. We examined a high volume of challenging surgical pathology cases. A subset of cases that Dr. Resident handed off to me ready for sign-out showed deficiencies related to diagnostic interpretation, details, and stylistic preparation of the pathology report below the expected level of performance for a resident at this level of training. Despite discussion during teaching rounds, these characteristics did not substantially improve by the end of the second week (confounding factors that could affect performance were not present). This fourth-year resident did not exhibit the credibility expected for their level of training and would benefit from a mentorship discussion about how their credibility will be questioned until they demonstrate improvement in these skills.

## Conclusion

In summary, the mission of the ACGME is to improve health care and population health by assessing and enhancing the quality of resident and fellow physicians' education through advancements in accreditation and education, and the ACGME Definition of GME reads in part: “Graduate medical education is the crucial step of professional development between medical school and autonomous clinical practice. It is in this vital phase of the continuum of medical education that residents learn to provide optimal patient care under the supervision of faculty members who not only instruct, but serve as role models of excellence, compassion, cultural sensitivity, professionalism, and scholarship”. One method to assist the ACGME mission is to produce physicians that have earned a respectable level of trust and credibility, as evidenced by their performance during GME training. While further investigation is required to evaluate if there is a benefit to the proposed credibility assessment in pathology GME, our roles as PDs, mentors, and GME leaders provide an opportunity and obligation to guide and nurture our residents and fellows toward the goal of acquiring trustworthiness and credibility upon the completion of their training.

## Declaration of generative AI and AI-assisted technologies in the writing process

During the preparation of this work, the author used ChatGPT in order to create Fig. 1. After using ChatGPT, the author reviewed and edited the content as needed and takes full responsibility for the content of the published article.

## Funding

This research received no specific grant from any funding agency in the public, commercial, or not-for-profit sectors.

## Declaration of competing interest

The Author declares that there are no competing interests.
